# Breast vibro-acoustography: initial experience in benign lesions

**DOI:** 10.1186/s12880-014-0040-1

**Published:** 2014-12-30

**Authors:** Azra Alizad, Mohammad Mehrmohammadi, Karthik Ghosh, Katrina N Glazebrook, Rickey E Carter, Leman Gunbery Karaberkmez, Dana H Whaley, Mostafa Fatemi

**Affiliations:** Department of Physiology and Biomedical Engineering, Mayo Clinic College of Medicine, 200 First Street SW, Rochester, MN 55905 USA; Division of General Internal Medicine, Department of Medicine, Mayo Clinic College of Medicine, 200 First Street SW, Rochester, MN 55905 USA; Department of Radiology, Mayo Clinic College of Medicine, 200 First Street SW, Rochester, MN 55905 USA; Division of Biomedical Statistics and Informatics, Department of Health Sciences Research Mayo Clinic College of Medicine, 200 First Street SW, Rochester, MN 55905 USA; Bolu IBD Hospital, Radiology, Sanayi Sitesi 32. Blok Demirciler Ve Nalburcular Odasi Hiz. Binasi Alti, No:1, 14100 Bolu, Turkey

**Keywords:** Breast neoplasms, Breast ultrasonography, Mammography, Vibro-acoustography, Benign breast lesion

## Abstract

**Background:**

Vibro-acoustography (VA) is a newly developed imaging technology that is based on low-frequency vibrations induced in the object by the radiation force of ultrasound. VA is sensitive to the dynamic characteristics of tissue. Here, we evaluate the performance of VA in identifying benign lesions and compare the results to those of mammography.

**Methods:**

An integrated mammography-VA system designed for in vivo breast imaging was tested on a group of female volunteers, age ≥ 18 years, with suspected breast lesions based on clinical examination. A set of VA scans was acquired after each corresponding mammography. Most lesions were classified as benign based on their histological results. However, in 4 cases, initial diagnosis based on clinical imaging determined that the lesions were cysts. These cysts were aspirated with needle aspiration and disappeared completely under direct ultrasound visualization. Therefore, no biopsies were performed on these cases and lesions were classified as benign based on clinical findings per clinical standards. To define the VA characteristics of benign breast masses, we adopted the features that are normally attributed to such masses in mammography. In a blinded assessment, three radiologists evaluated the VA images independently. The diagnostic accuracy of VA for detection of benign lesions was assessed by comparing the reviewers’ evaluations with clinical data.

**Results:**

Out of a total 29 benign lesions in the group, the reviewers were able to locate all lesions on VA images and mammography, 100% with (95% confidence interval (CI): 88% to 100%). Two reviewers were also able to correctly classify 83% (95% CI: 65% to 92%), and the third reviewer 86% (95% CI: 65% to 95%) of lesions, as benign on VA images and 86% (95% CI: 69% to 95%) on mammography.

**Conclusions:**

The results suggest that the mammographic characteristics of benign lesion may also be used to identify such lesions in VA. Furthermore, the results show the ability of VA to detect benign breast abnormalities with a performance comparable to mammography. Therefore, the VA technology has the potential to be utilized as a complementary tool for breast imaging applications. Additional studies are needed to compare the capabilities of VA and traditional ultrasound imaging.

## Background

There are several different medical imaging modalities for the screening and diagnosis of breast cancer [1-3]. However, benign breast lesions are much more common than malignant lesions, and accurate diagnosis of these lesions is important for optimal care of the patient [4]. Benign breast disease (BBD) is a well-known, significant risk factor for breast cancer [5]. BBD is diagnosed when a woman has a breast biopsy for a palpable or imaging abnormality in her breast that results in benign findings.

Any imaging evaluation of the breast with high sensitivity may also be associated with increased false positive results that may leads to unnecessary (i.e. benign) biopsies, resulting in high cost as well as significant trauma and anxiety for the patients due to this invasive procedure and in some instances, they may choose unnecessary extensive varieties of surgeries such as mastectomy [6,7]. Moreover, accurate identification of benign non-proliferative conditions such as cysts and fibroadenomas can reduce unwanted benign breast biopsies. It is, therefore, important to develop imaging tools with higher specificity to reduce false positive test results. Mammographic breast density (MBD), another known factor, reduces the sensitivity of mammograms [8]. Hence, there is an immense need for a noninvasive tool that can assess breast tissue characteristics while not influenced by MBD.

Conventional breast ultrasonography (US) is routinely used as an adjunct imaging tool to x-ray mammography for diagnosis of breast pathology; it improves sensitivity and has a considerable role in differentiation of cysts and solid nodules with higher specificity. US can also help to characterize solid breast masses [3,9-14]. US features such as lesion shape, orientation to the skin line, lesion boundary, margin characteristics, echo-pattern, posterior acoustic appearance, and effects on the surrounding breast tissue are employed to reach a Breast Imaging and Reporting Data System (BI-RADS) assessment [15]. In BI-RADS, the lesions are categorized into 7 categories numbered 0 to 6. Categories 1 to 3 indicate being negative, benign findings, or probably benign, respectively. Categories 4 and 5 are suspicious for malignancy, and 6 refers to having known biopsy proven malignancy [15]. Abnormalities on screening mammography that require further evaluation are assessed as category 0; similarly, calcifications found on breast US are also in category 0 assessment [16]. US is usually used for the subsequent evaluation of BI-RADS category 0 mammograms [17].

Benign breast lesions have a characteristic sonographic appearance; cysts appear as well-circumscribed, round or oval anechoic or hypoechoic masses with unnoticeable walls and posterior acoustic enhancement [18]. Fibroadenomas typically appear as oval, well-circumscribed masses with an abrupt interface and homogeneous iso- or hypoechoic echo texture. Benign papilloma masses appear as solid masses within a dilated duct with a vascular feeding pedicle seen on color Doppler imaging [2,9,14]. There is, however, an overlap between benign and malignant lesion US characteristics leading to a significant number of false-positive cases, which are recommended for US-guided percutaneous biopsy [14]. An additional sonographic tool to help decrease the number of unnecessary biopsies can play an important role to reduce such a large number of unnecessary biopsies. Imaging modalities, particularly those that provide palpation-like information, can help to better diagnose and identify breast lesions.

Elasticity imaging is an emerging field of medical imaging that provides such information. Elasticity imaging consists of magnetic resonance elastography (MRE) [23,24] which is expensive and not widely available [19,20], conventional quasi-static ultrasound elastography [21-23], Acoustic Radiation Force Impulse (ARFI) imaging [24-26] and Shear Wave Elastography (SWE, also called SuperSonic Imaging (SSI)) [27-31]. ARFI and SWE use ultrasound radiation force to generate shear waves and quantify tissue elasticity from measured propagation speed of shear waves [32,33]. The results of studies using ARFI and SWE for breast have been very promising.

Vibro-acoustography (VA) is an imaging modality that also provides palpation-like information [34]. VA is introduced as a complementary technique to improve sensitivity and specificity in clinical breast imaging [35]. Principles of VA have been described extensively [34,36-39]. In VA, ultrasound is employed to produce a localized low-frequency force to vibrate the tissue. In technical terms, such a force is called “acoustic radiation force (ARF)”. The low-frequency ARF is generated by two intersecting continuous wave (CW) focused ultrasound beams at slightly different frequencies. This force, which acts as a point force, vibrates the object at a frequency equal to the difference between two US frequencies (typically in kHz range). The resulting vibrations produce an acoustic emission field that is detected by a sensitive microphone (or hydrophone). Harder tissues normally produce a significantly different acoustic emission compared to normal soft tissues. Conceptually, VA resembles palpation; i.e., detects tissue response to an exerted force on tissue. [34,36]. However, VA benefits from a significant advantage of using a highly localized ARF, which leads to the possibility of assessing tissue properties on a small scale. As a result, VA can provide detailed information on tissue mechanics at high resolution [38,39].

Compared to conventional US imaging, VA images are speckle free, thus, the images have high-contrast that allows detection of small structures [4,40,41]. Thus VA can be a complementary tool to the existing breast imaging tools. Compared to elastography techniques, VA uses dynamic acoustic radiation force in the range of 10s of kHz, which is much higher than the frequency used in quasi-static elastography, ARFI, and shear wave imaging (normally in 10s to 100s Hz range). VA mages represent the acoustic response of tissue, which is a complex function of several parameters, including the elasticity and viscosity. However, elasticity cannot be directly and quantitatively measured from this acoustic response.

Our preliminary studies demonstrated the abilities of VA imaging in various tissues [40,42,43] including *in vivo* human breast [35,44]. In our previous studies [35,44], we used a confocal VA system combined with mammography to image various breast abnormalities, [35]. Since VA is a new modality, characteristic of different types of lesions (benign or malignant) in VA are not necessarily known. A goal of this paper is to determine and present VA characteristics of benign breast lesions. Another goal of this paper is to evaluate the performance of such characteristics in identifying benign lesions in VA and compare the results and compare the results to those of mammography. To define the VA characteristics of benign breast masses, we adopted the features that are normally attributed to such masses in mammography. Such features, which are often defined in contrast to those of malignant masses, include morphological attributes of the mass and information related to calcifications. We will test the validity and performance of these characteristics in a reader-based study.

## Methods and materials

### Study subjects

Under an approved protocol by the Mayo Clinic Institutional Review Board (IRB), female volunteers (18 and up) were chosen for the study and informed consent was obtained from all enrolled patients. Pregnant women were excluded from this study. We selected 36 patients with benign lesions based on pathology and clinical data. Five of the 36 patients examined were used for training and excluded from the study. Also, two participants were excluded from the study because of accidental hardware failure that occurred during the study. In the group of 29 women with benign lesions, 25 underwent ultrasound-guided core needle biopsy. The other 4 cases, based on radiologist impression of mammography and/or breast ultrasound, diagnosis of simple cyst was made and the cysts were aspirated and disappeared completely under direct ultrasound visualization, therefore, no histology was necessary for these 4 cystic lesions.

### VA system

**Figure 1:**
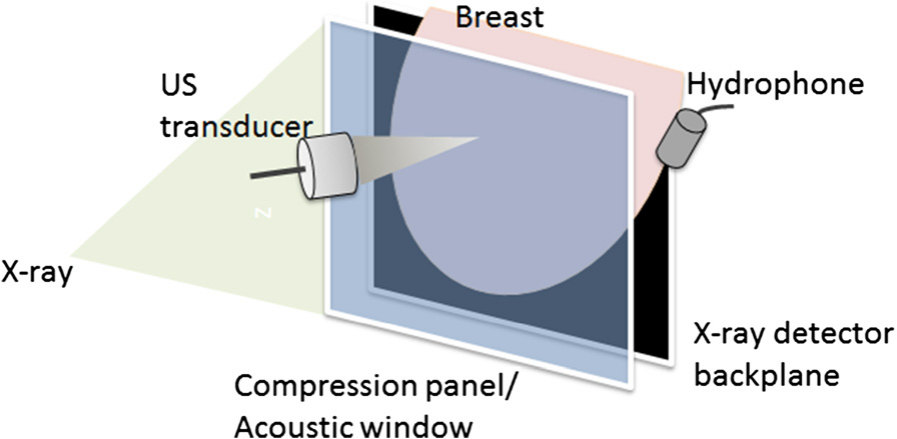
**Schematic of a VA system combined with mammography illustrating the breast position.** During mammography, the transducer and hydrophone are transferred out of the x-ray path. A compression panel is covered with a latex membrane with the size of 80 × 80 mm acoustic window. The imaging area within the acoustic window is 50 × 50 mm.

An experimental mammography-VA system, designed for *in vivo* breast imaging [35], was used to image patients with benign breast lesions. The VA system was integrated into a clinical stereotactic mammography machine (MammoTest system; Fischer Imaging, Inc, Denver, Colorado, USA) so that we could have matching VA and mammography images (from the same view angle) for comparison. Figure 1 represents the diagram of this system. It should be noted that VA is a noninvasive imaging tool, and it has been shown that VA can function at ultrasound intensities within the FDA limits. VA System parameters are: transducer frequency = 3 MHz, transverse resolution = 0.7 mm, scanning increments = 0.2mm, ultrasound intensity at the focal point = 700 mW/cm^2^ in compliance with the FDA recommendation for *in vivo* diagnostic ultrasound [45]. The thermal safety of VA system is discussed in detail in [46].

The patient rested on an examination bed in a prone position. Through a hole in the bed, the breast was positioned between the back panel, including an x-ray detector, and a sliding panel that slightly compresses the breast. The compression was constant and at minimal level during VA acquisitions. A thin latex membrane covers the window of the compression panel that is transparent to the US beams, and the US transducer is located behind the window. All VA images were acquired in the cranial-caudal view at various depths from the skin. The VA images were acquired by mechanically scanning the confocal VA probe, and each scan covered a 50 × 50 mm area with a scan step size of 200 μm in each direction. VA image resolution is determined by the spatial resolution of the mixed US beams (i.e. low frequency ARF) and was about 700 μm [34,47]. A hydrophone (Bruel & Kjaer model 8106) was placed on the side of the breast to receive the acoustic emission generated by the radiation force of US. Upon finishing a mammography scan, a set of VA scans at different depths was acquired by adjusting the distance between the confocal VA probe and breast tissue.

### Reference standard documentation

After mammography, a set of VA scans was acquired by the experimental device. Then we selected the group of patients with benign lesions based on pathology, and in some cases, based on clinical findings and radiologist impression. To characterize the benign lesions’ morphology, we reviewed all data including the corresponding X-ray mammograms acquired during the VA testing, other available clinical images such as clinical mammograms, US, and clinical data such as palpation information from the patient record. Based on these data, the shape and location of the benign lesion in the VA imaging window were determined.

### Criteria for benign and malignant masses

Findings such as irregular or oval shaped; indistinct or ill-defined borders, presence of architectural distortion and or spiculations, presence of clustered pleomorphic microcalcifications are common characteristics of malignant breast masses. Round punctate calcifications are mostly benign unless appears as a segmental or linear distribution would be at least suspicious [48]. Other findings such as circumscribed and distinct borders, round shape and lobulated masses, or simple cysts with soft wall are characteristics of benign lesions. However, *intra cystic mass,* masses with eccentric cystic spaces or thick wall cysts with thick separation are usually malignant [18].

### Reader interpretation

VA images were evaluated independently by three independent reviewers (radiology residents) who identified breast lesions and location. Because the reviewers did not have prior experience with VA imaging interpretation, they underwent a training session to learn about VA images and familiarize themselves with the general appearance of normal breast tissues. Masses and calcification in a typical VA image and five images used for training were excluded from the study. For training purposes, all the clinical data were given to the reviewers. However, for the remaining test set, we asked reviewers to locate and identify the benign lesion without having access to any clinical data. The reviewer evaluated each lesion based on the criteria for the benign masses including size, shape, margins, presence of microcalcifications, and presence of architectural distortion and/or spiculations, and determined if lesion was benign or not. In a separate session, the reviewers evaluated only the mammography images based on similar criteria.

### Statistical considerations

Observer performance for detection of a breast lesion and correct classification of the lesion was measured using proportions and score confidence intervals.

## Results

**Figure 2:**
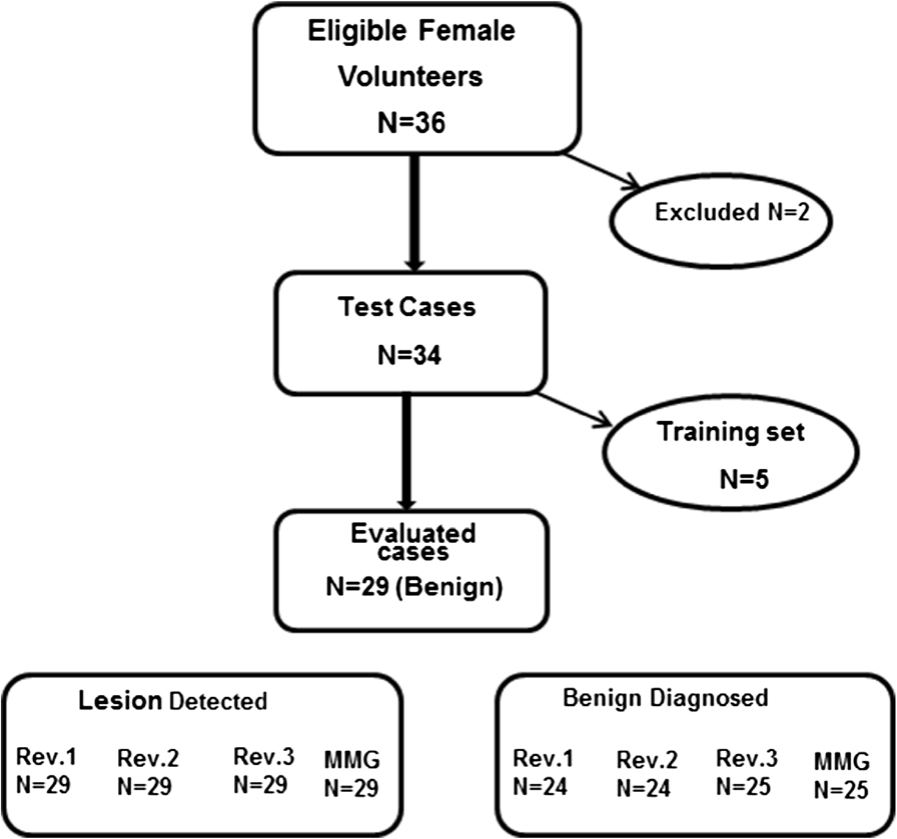
**Flow chart indicating the result of VA image review by three radiologists aiming on identification of benign abnormalities.** (MMG = mammography, Rev = Reviewer).

Women volunteers with abnormality in their clinical breast examination and/or mammography BI-RADS category 4 or less were included in this study. All patients underwent clinical mammography and breast US before participation in the study. VA imaging was done on all subjects. In total, 36 patients with benign lesions were evaluated; five were used for training and excluded from the evaluation. Also, two participants were excluded from the study because of accidental hardware failure that occurred during the study. A total of 29 patients, averaging 44 years old, with benign lesions were evaluated. The final diagnosis for 25 patients was based on histological results. In the remaining 4 patients, based on clinical imaging, the lesions were determined to be simple cysts. These cysts were aspirated with needle aspiration and disappeared completely under direct ultrasound visualization. Therefore, the final diagnosis includes six benign cysts, 15 fibroadenomas, three papillomas, and three of post-surgical scar tissue, and two focal atypical ductal hyperplasia with microcalcifications. The flow chart shown in Figure 2 demonstrates the results of VA image evaluation by three independent readers’ reviews in 29 patients with benign lesions and their final diagnosis. The lesion detection rate for each radiologist was 100% with (95% confidence interval (CI): 88% to 100%). While all 29 lesions were confirmed to be benign (25 biopsy proven and 4 cysts with aspiration), the primary clinical imaging review of reference image (mammography) indicated that four lesions were suspicious for malignancy, therefore, the correct classification as benign was 86% (95% CI: 69% to 95%). This uncertainty was also observed using the new imaging modality. Correct classification as benign by the reviewers was either 83% (95% CI: 65% to 92%) or 86% (95% CI: 69% to 95%) as shown in Figure 2. Readers 1 and 2 misclassified three of the lesions as suspicious and two as malignant. Reader 3 misclassified three of the lesions as suspicious and one as malignant. These misclassified cases are discussed in the following section.

### Review of select cases

To further demonstrate the abilities of VA in detecting breast benign lesions, we present VA images of eight identified cases and compare VA results with that of conventional US and mammography. We also present three of the cases that were misclassified in mammography as well as VA images.

#### Cases of fibroadenomas identified by both mammography and VA

##### Case 1

**Figure 3:**
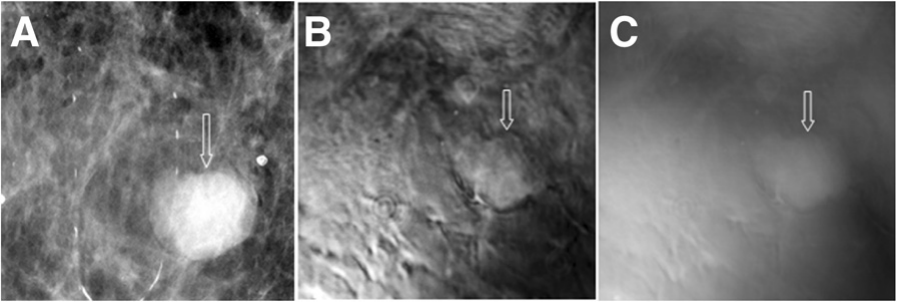
**VA and mammography images of Case 1.** **(A)** Prone cranial-caudal mammogram of the right breast shows a mass. **(B)** and **(C)** are VA images at 50 and 20 KHz frequencies, respectively, at a depth of 25 mm. The structural details are more pronounced at 50 kHz. The arrows mark the location of the mass. The slight upward shift was due to patient movement after mammography.

The patient was a woman in her 70s. Her screening mammography showed scattered fibroglandular densities in both breasts and a mass lesion in her right breast (fibroadenoma). The prone cranial-caudal mammogram of the right breast showed a 2 cm, sharply marginated mass with coarse lobulations of a soft-tissue mass (Figure 3A). The fibroadenoma region was clearly seen in the VA images, taken at a depth of 25 mm below the skin (Figure 3B and C), denoted by arrows. The VA images could show the gentle coarse lobulation in the mass, a classic finding in fibroadenoma, and the margin very well. The mammogram additionally showed a well-circumscribed 3 mm calcification near the mass, but it was out of focus in the VA image, due to its different depth (Figure 3). This case demonstrates that VA can identify fibroadenomas.

#### Cases of fibroadenomas not seen on mammography but identified on VA

##### Case 2

**Figure 4:**
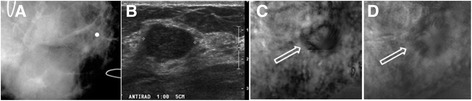
**VA, US and mammography images of Case 2.** **(A)** Prone cranial caudal mammogram image corresponding to the VA image showing only a marker placed in the vicinity of a palpable mass. **(B)** US of same breast shows a hypoechoic lobulated mass. **(C and D)** VA images of the same breast at 60 kHz and at 20 mm and 25 mm depths, respectively. Arrows mark the location of the breast mass.

The patient, a woman in her 40s, presented with a palpable mass in the right breast. Targeted US showed a 29 × 19 × 13 mm well-defined lobulated and mildly hypoechoic mass. Her diagnostic mammography showed heterogeneous dense parenchyma in both breasts, but was not able to detect the palpable mass on right breast. A marker was placed on the skin to identify the approximate location of palpable mass as seen in the ultrasound (Figure 4A and B). The VA images indicated a round mass with a defined border and some lobulation inside denoted by arrows (Figure 4C and D). The pathology result revealed the mass to be a fibroadenoma. This case demonstrates that VA can identify mass lesions not seen on mammograms.

##### Case 3

**Figure 5:**
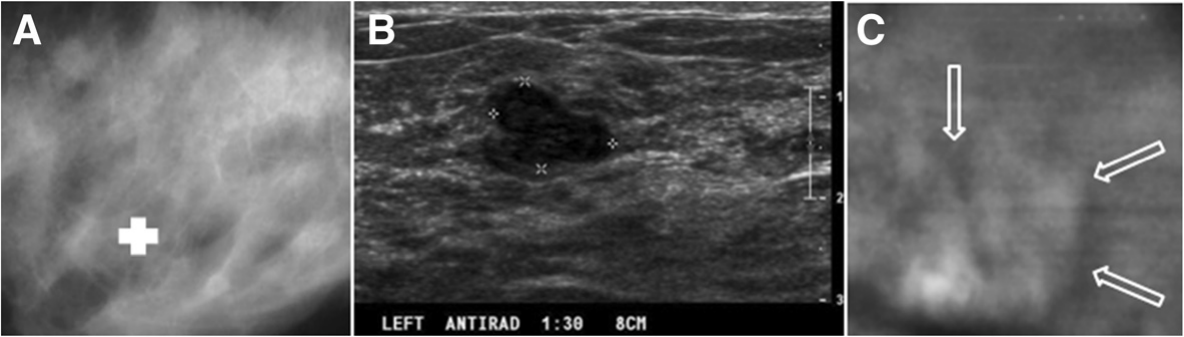
**VA, US and mammography images of Case 3.** **(A)** Mammography does not show any discrete abnormality, the lesion location marked by our radiologist. **(B)** US image of left breast shows a hypoechoic mass. **(C)** VA image shows a lobulated mass in an area of concern marked by our radiologist.

The patient, in her 30s, presented with a palpable lesion in the left breast. Her mammography showed an extremely dense breast, and there is no discrete mammographic abnormality in the area of the palpable abnormality because of the highly dense tissue. The US image showed a benign appearing hypoechoic mass in the left breast. The VA image reveals a lobulated mass with a defined border (Figure 5). This case demonstrates that VA can identify mass lesions (in this case a biopsy proven fibroadenoma) not seen on mammograms.

##### Case 4 (Benign Papilloma)

**Figure 6:**
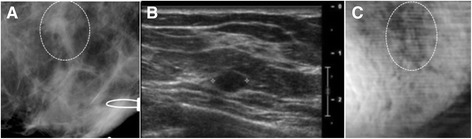
**VA, US and mammography images of Case 4.** **(A)** X-ray mammogram of the left breast, which corresponds to the same view as VA showing a small nodular density. **(B)** US of left breast shows 1 cm elongated solid circumscribed nodule. **(C)** VA image at a 30 mm depth shows the elongated solid nodule at the same location of X-ray.

The patient was in her 50s. Her mammography demonstrated scattered fibroglandular density on both breasts and a small nodular density measuring about 10 mm in her left breast. Clinical US revealed a 10 mm elongated solid circumscribed nodule. VA images acquired at a 30 mm depth showed the same elongated nodule at the same location as that shown in X-ray (Figure 6). The result of pathology classified the lesion as a papilloma with stromal fibrosis. This case demonstrates the ability of VA in identifying a benign papilloma.

#### Cases of benign cysts

##### Case 5

**Figure 7:**
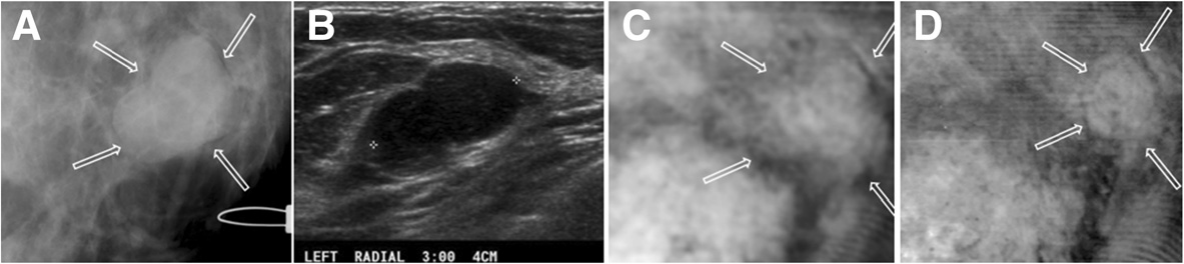
**VA, US and mammography images of Case 5.** **(A)** Mammogram showing a 20 mm circumscribed lobulated mass. **(B)** Targeted US showing a hypoechoic mass. **(C)** and **(D)** are VA images at depths of 30 mm and 35 mm, respectively. A mass with soft border is observed at the same location as the mammogram (denoted by arrows).

The patient, in her 50s, presented with a palpable mass in the left breast. Her mammography demonstrated scattered fibroglandular densities (D2) in both breasts and a circumscribed lobulated mass measuring about 2 cm in the left breast. Targeted US indicated a benign cyst in the area comparable to mammography. VA images at a different depth (Figure 7) showed a well-defined mass about the same size and location seen in the mammogram (denoted by arrows). The patient was diagnosed with a benign cyst and the fluid was completely drained under guided US. This case demonstrates that VA can identify benign cystic lesions.

##### Case 6

**Figure 8:**
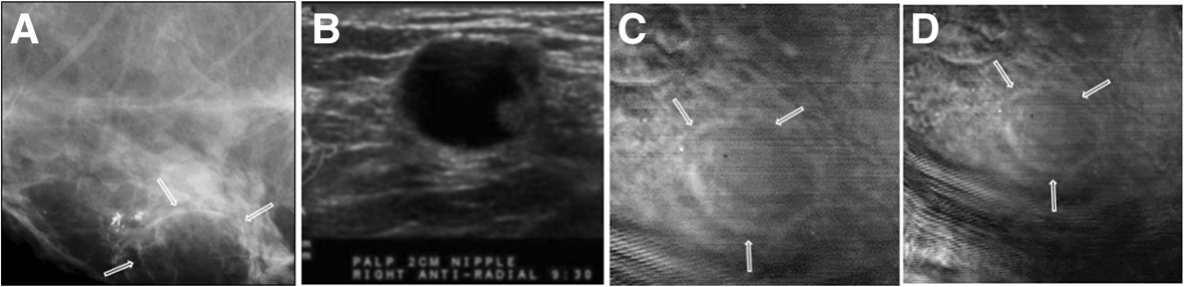
**VA, US and mammography images of Case 6.** **(A)** Mammogram taken corresponding to the VA view showing a 20 mm well-circumscribed lesion with soft border and several calcifications near the lesion. **(B)** Targeted US showing a hypoechoic mass confirmed as an oil cyst. **(C and D)** are VA images at 25 mm and 30 mm depths, respectively. The well-defined mass with soft border is seen in all VA images. Note: breast repositioned in **(D)** and cyst seen in center of the image.

The patient, in her 40s, presented with an oil cyst in the right breast near the nipple. Mammography demonstrated scattered fibroglandular tissue and a 20 mm radiolucent well-defined mass with soft border, which was consistent with an oil cyst, along with several calcifications near the larger cyst. Clinical US revealed the presence of the cyst as a hypoechoic mass. VA images acquired at 20, 25, and 30 mm depths (Figure 8) confirmed the presence of a well-defined circumscribed mass with soft border. This case demonstrates that VA can identify a benign oil cyst.

#### Misidentified cases

##### Case 7

**Figure 9:**
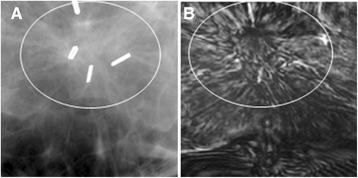
**VA and mammography images of Case 7.** **(A)** X-ray mammogram of left breast which corresponds to the same view as VA showing a radial pattern architectural distortion with surgical clips. **(B)** VA image at 20 mm depth showing the same speculated-like architectural distortion denoted by the circle. Parts of surgical clips can also be seen in the VA images.

The patient, in her 50s, presented with a previous lumpectomy site above the left nipple. The patient was initially scheduled for a screening mammography, but a questionable new abnormality on the left breast necessitated additional imaging evaluation. Diagnostic mammogram demonstrated scattered fibroglandular densities in both breasts, and architectural distortion in a predominately radial pattern that could be due to resection of an invasive carcinoma approximately 5 years earlier or recurrence of cancer. VA images showed the area of concern as a spiculated or radial pattern architectural distortion (Figure 9). Final assessment was benign post-operative changes.

We had three cases of post-surgical scar tissue that imitated spiculation, the hallmark of a cancerous lesion; one shown in Figure 9. These cases were misidentified as malignant in both mammography and VA because of the misleading radial pattern distortion due to post-surgical scar tissue.

##### Case 8

**Figure 10:**
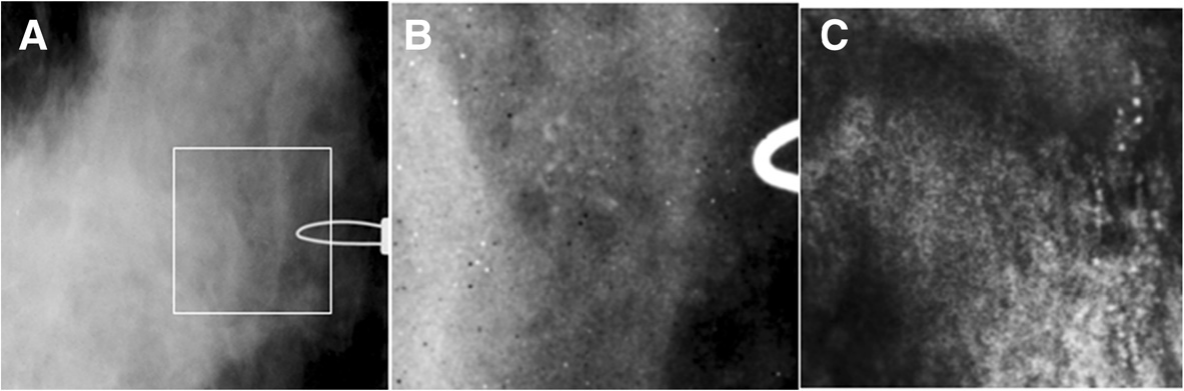
**VA and mammography images of Case 8.** **(A)** A cluster of calcifications and some scattered microcalcifications, which can be better seen in the magnified images of the square box part of x-ray shown in **(B)**. **(C)** VA image of the breast at 1 cm depth and normal sum of frequencies. The cluster of microcalcifications and ductal calcification are visible. Note: Due to patient’s repositioning during the experiment, calcifications are seen on the far right of the VA image.

The patient was in her early 50s. Her mammography shows extremely dense breasts. Diagnostic mammography demonstrated indeterminate calcifications, clustered and scattered, in the left breast. VA was also able to reveal this cluster of microcalcifications and ductal calcifications. Biopsy presented focal atypical ductal hyperplasia (ADH) with associated calcifications (Figure 10). This patient had an extremely dense breast and due to indeterminate calcifications, both mammography and VA misidentified this case as malignant.

## Discussion

The goal of this study was to primarily investigate the diagnostic accuracy of VA in detecting benign breast lesions. Various types of benign breast lesions including lipid cyst, benign cyst, benign fibroadenoma, and benign papilloma were studied with a laboratory-built VA system integrated with a mammography machine. The results of the study clearly show the efficacy of VA in detecting benign breast lesions. Also, the results show that the mammographic attributes of benign lesions can be adopted to identify such lesions in VA imaging.

We explored the VA characteristics of a wide range of benign lesions. Regular shape, well-defined lesion boundary, distinct or soft margin, and soft lobulation are the most benign characteristics we found on VA images. Cysts appeared on VA images as well-defined masses with a soft border as seen in Figures 7 and 8 and all reviewers could identify all cyst cases. Fibroadenomas, the most common solid breast masses that undergo breast biopsy, appeared on VA images as well-circumscribed ovoid or round masses with gentle lobulation, and in some cases were calcified as seen in US and mammography, which happens in degenerating fibroadenomas [49,50].

Three cases of fibroadenomas and one case of papilloma that were unidentifiable by mammography were marked by our radiologist and detected by VA. Similarly seen in breast US [14], VA can help identify lesions that are obscured in mammography due to dense breast. VA also identified benign papilloma lesions as elongated nodules in a dilated duct; one papilloma was not identifiable on mammography but seen using VA. Diagnosis of papilloma is important and surgical excision should be considered for core needle biopsy proven benign papilloma - especially for lesions larger than 1.5 cm, regardless of imaging findings [41]. As expected, VA worked better than mammography in dense breast.

The cases that were misclassified by VA were also misidentified by mammography. Three of the misidentified cases were post-surgical scar tissue, as shown in Figure 9, presenting as a radial pattern distortion imitating spiculation, which is most seen in malignant tissue. It should be noted that mammographically only some of these changes can be differentiated based on morphological characteristics. Even though microcalcifications are often associated with breast carcinoma, not all spiculated lesions including microcalcifications are considered malignant. Mammography alone is often not a reliable imaging tool for making the definitive diagnosis in these cases. Additional mammographic views, breast US, clinical breast examination, and needle or surgical biopsy are often required [18].

Two other misclassifications occurred in focal atypical ductal hyperplasia (ADH) with associated calcifications as shown in Figure 10. The presence of a cluster of indeterminate microcalcifications is always suspicious of ADH or ductal carcinoma in situ (DCIS). ADH is a lesion with significant malignant potential. The diagnosis of ADH at needle core breast biopsy is normally considered as an indication for surgical excision [19,21,46]. In these two cases, VA and mammography were in agreement on suspicious lesions.

The VA images represented in the results section are simply reconstructed from the amplitude of the acoustic emission; additional signal and image processing algorithms were not required. The resolution of the VA images is determined by the spatial distribution of the ARF and is in the sub-millimeter range. Such resolution is generally sufficient for detecting the breast lesions. Since VA and US share common hardware (US scanner and transducer), we envision that in the future VA can be combined with conventional US imaging and become a hybrid imaging modality that can provide physicians with further clinically useful information.

We used mammography as the reference modality because the confocal VA system is integrated with a mammography machine that allowed acquiring matching mammography and VA images from the same view angle and through the same imaging window, which facilitated the comparison. We also note that traditional breast US and elastography images are obtained in the B-Plane, which is perpendicular to the C-plane images of VA. This difference in imaging plane makes it difficult to compare VA against US and elastography images to match the location of breast lesions. In contrast, we can obtain VA and mammography from an identical imaging window, thus one-to-one comparison becomes possible. We also emphasize that VA is introduced as a complementary technique to improve sensitivity and specificity in clinical breast imaging [35]. Compared to conventional US imaging, VA images are speckle free, thus, the images have high-contrast that allows detection of small structures such as microcalcifications. Thus VA can be a complementary tool to the existing breast imaging tools. Compared to elastography techniques such as ARFI [32] and SWI [33], VA uses dynamic acoustic radiation force in the range of 10s of kHz [34], which is much higher than the frequency used in quasi-static elastography, ARFI, and shear wave imaging (normally in 10s to 100s Hz range). VA images represent the acoustic response of tissue, which is a complex function of several parameters, including the elasticity and viscosity. However, elasticity cannot be directly and quantitatively measured from this acoustic response.

VA imaging of the breast with a confocal VA system in the prone position has certain limitations. One of the drawbacks of this system is limited access to parts of the breast near the chest wall. This was due to mechanical limitations caused by the patient position (prone position) which put a part of the breast close to the chest wall outside the imaging window and thus not accessible. Also, the need for two-dimensional raster scanning of the transducer resulted in slow image acquisition. To overcome such limitations of confocal VA, we recently developed a new VA system with a handheld array transducer that is implemented on a clinical ultrasound scanner. This system is now being tested in an ongoing study on patients in supine position [51].

We understand that malignant lesions are clinically more important. We decided to focus this study on benign breast lesions, because benign lesions are also clinically important as they are much more common than malignant breast lesions. Benign breast lesions cover a wide range of abnormalities with different VA characteristic, thus warranting a separate a study.

Furthermore, the case selection did not represent a typical screening process. Nonetheless, this study represented an important first step in studying the utility of VA for detection and characterization of breast masses. Despite the mentioned limitations, the results of this study show the ability of the technique to identify the benign lesions with high clarity and present VA characteristics of a wide range of benign lesions.

Although the sample size in the present study was rather small, we believe that our results can be reproduced and validated in future clinical studies. We anticipate that by implementing VA on a clinical US scanner and by using a 2D US transducer [52], VA will become a more suitable tool for clinical usage. Additional studies are needed to compare the capabilities of VA and traditional ultrasound imaging.

### Consent

Written informed consent was obtained from the patient(s) for publication of this manuscript and accompanying images.

## Abbreviations

BBD: Benign breast diseases
ARF: Acoustic radiation force
VA: Vibro-acoustography
US: Ultrasonographic, ultrasonography, ultrasound
SWE: Shear wave elastography
SWI: Shear wave imaging
SSI: Supersonic imaging
ARFI: Acoustic radiation force impulse
